# Tumor immune contexture predicts recurrence after prostatectomy and efficacy of androgen deprivation and immunotherapy in prostate cancer

**DOI:** 10.1186/s12967-022-03827-4

**Published:** 2023-03-14

**Authors:** Sujun Han, Taoping Shi, Yuchen Liao, Dong Chen, Feiya Yang, Mingshuai Wang, Jing Ma, Hu Li, Yu Xu, Tengfei Zhu, Wenxi Chen, Guoqiang Wang, Yusheng Han, Chunwei Xu, Wenxian Wang, Shangli Cai, Xu Zhang, Nianzeng Xing

**Affiliations:** 1 Department of Urology, National Cancer Center/National Clinical Research Center for Cancer/Cancer Hospital, Chinese Academy of Medical Sciences and Peking Union Medical College, No.17 Panjiayuan Nanli, Chaoyang District, Beijing, 100021 China; 2 Department of Urology, Chinese PLA General Hospital, No 28 Fuxing Road, Beijing, 100853 China; 3 Burning Rock Biotech, Guangzhou, 510300 China; 4 Department of Urology, Shanxian Central Hospital of Shandong Province, Heze, 274300 Shandong China; 5 Institute of Basic Medicine and Cancer (IBMC), Chinese Academy of Sciences, Hangzhou, 310022 China; 6 Department of Clinical Trial, The Cancer Hospital of the University of Chinese Academy of Sciences (Zhejiang Cancer Hospital), Hangzhou, 310022 China

**Keywords:** Prostate cancer, Tumor immune microenvironment, Biochemical recurrence, Androgen deprivation therapy, Immunotherapy

## Abstract

**Background:**

Prostate cancer is one of the most common cancers in men with notable interpatient heterogeneity. Implications of the immune microenvironment in predicting the biochemical recurrence-free survival (BCRFS) after radical prostatectomy and the efficacy of systemic therapies in prostate cancer remain ambiguous.

**Methods:**

The tumor immune contexture score (TICS) involving eight immune contexture-related signatures was developed using seven cohorts of 1120 patients treated with radical prostatectomy (training: GSE46602, GSE54460, GSE70769, and GSE94767; validation: GSE70768, DKFZ2018, and TCGA). The association between the TICS and treatment efficacy was investigated in GSE111177 (androgen deprivation therapy [ADT]) and EGAS00001004050 (ipilimumab).

**Results:**

A high TICS was associated with prolonged BCRFS after radical prostatectomy in the training (HR = 0.32, 95% CI 0.24–0.45, *P* < 0.001) and the validation cohorts (HR = 0.45, 95% CI 0.32–0.62, *P* < 0.001). The TICS showed stable prognostic power independent of tumor stage, surgical margin, pre-treatment prostatic specific antigen (PSA), and Gleason score (multivariable HR = 0.50, 95% CI 0.39–0.63, *P* < 0.001). Adding the TICS into the prognostic model constructed using clinicopathological features significantly improved its 1/2/3/4/5-year area under curve (*P* < 0.05). A low TICS was associated with high homologous recombination deficiency scores, abnormally activated pathways concerning DNA replication, cell cycle, steroid hormone biosynthesis, and drug metabolism, and fewer tumor-infiltrating immune cells (P < 0.05). The patients with a high TICS had favorable BCRFS with ADT (HR = 0.25, 95% CI 0.06–0.99, *P* = 0.034) or ipilimumab monotherapy (HR = 0.23, 95% CI 0.06–0.81, *P* = 0.012).

**Conclusions:**

Our study delineates the associations of tumor immune contexture with molecular features, recurrence after radical prostatectomy, and the efficacy of ADT and immunotherapy. The TICS may improve the existing risk stratification systems and serve as a patient-selection tool for ADT and immunotherapy in prostate cancer.

**Supplementary Information:**

The online version contains supplementary material available at 10.1186/s12967-022-03827-4

## Introduction

Prostate cancer is a malignant tumor with high incidence and mortality worldwide [1]. It is characterized by a long and varied course of the disease and high intra- and inter-tumor heterogeneity [2]. Prostate cancer is an androgen-dependent disease, and most prostate cancer patients initially respond to androgen deprivation therapy (ADT) [3]. However, most androgen-sensitive prostate cancer patients eventually become resistant to ADT and develop castration-resistant prostate cancer (CRPC) [4].

The tumor immune microenvironment (TIME) is mainly composed of tumor cells, immune cells, various signaling molecules, and extracellular matrix [5]. TIME was closely associated with survival outcome and treatment response [6]. Several pieces of evidence have indicated that TIME plays a key role in the development of prostate cancer and the response to anti-tumor therapy [7, 8]. T-cell infiltration was related to tumor progression and cancer-specific survival in both localized and metastatic prostate cancer patients [9, 10]. For instance, T helper (Th) cells activated antigen-specific effector cells and recruited immune cells such as macrophages and mast cells, which could play crucial roles in the adaptive immune response to tumor cells [11, 12]. Th17 cells are a subgroup of CD4^+^ helper T cells, which can secrete interleukin (IL)-17 and IL-22, and play a critical role in the occurrence and development of prostate cancer [13]. The preponderance of Th17-mediated inflammation has been negatively correlated with Gleason score in prostate cancer patients [14], and blocking the IL-17 pathway inhibited the formation of microinvasive prostate cancer in animal models [15]. Given this, we speculated that TIME might be used to predict the biochemical recurrence-free survival (BCRFS) of prostate cancer.

As for immunotherapy, the anti-tumor effects of pembrolizumab and ipilimumab against prostate cancer have been demonstrated in clinical trials [16–18]. Immune checkpoint inhibitors (ICIs) such as the antibodies targeting cytotoxic T lymphocyte antigen-4 (CTLA-4) and programmed cell death 1 (PD-1)/programmed cell death-ligand 1 (PD-L1) could regulate the TIME and influence immune cells to promote anti-tumor immune responses [17, 19]. TIME might presumably also be a predictor for the ICI-induced antitumor activity in prostate cancer.

In this study, the multi-dimensional data of 1120 prostate cancer patients from The Cancer Genome Atlas-prostate adenocarcinoma (TCGA-PRAD) cohort and 6 Gene Expression Omnibus (GEO) cohorts were analyzed. In the training cohorts, we first evaluated the prognostic effects of 92 immune contexture-related signatures and constructed a tumor immune contexture score (TICS) based on the 8 signatures with prognostic effects. Then, the TICS was tested successfully in the validation cohorts. The TICS showed consistent prognostic effects across the prostate cancer patients with different clinicopathological characteristics (e.g., TNM stage, pre-treatment prostatic specific antigen [PSA], and Gleason score) and this score was an independent prognostic factor of BCRFS. Compared with the prognostic model constructed by the clinicopathological features without the TICS, the model with the TICS had a significantly higher power of prognostication. In addition, the correlations between the TICS with cancer-related signaling pathways and immunotherapy efficacy were also explored, which might provide promising insight into the individualized treatment of prostate cancer patients.

## Methods

### Prostate cancer datasets and pre-processing

Transcriptome profiles and clinicopathological characteristics of seven datasets of localized prostate cancer were downloaded from TCGA and GEO databases, including GSE46602, GSE54460, GSE70768, GSE70769, GSE94767, DKFZ2018, and TCGA-PRAD [20–24]. The Affymetrix microarray data from GSE46602 and GSE94767 were normalized using the robust multiarray analysis (RMA) algorithm. The Illumina microarray data from GSE70768 and GSE70769 were normalized using the quantile algorithm and log_2_ transformed. The RNA-seq data of GSE54460, DKFZ2018, TCGA-PRAD, GSE111177, and EGAS00001004050 produced by the Illumina HiSeq or TruSeq platform were normalized as Fragments Per Kilobase of exon model per Million mapped fragments (FPKM) or Reads Per Kilobase per Million mapped reads (RPKM) format and were transformed to log_2_ scale (for details, see Additional file 1: Table S1). The patients who had not received radical prostatectomy (RP) or had no BCRFS data were excluded. The seven PRAD datasets were split into the training set and the validation set based on techniques and platforms, ensuring that the training and validation sets each have datasets using microarray and RNA-seq (Additional file 1: Table S1).

To assess the utility of the TICS in predicting outcomes of patients receiving ADT, a cohort consisting of the pre-ADT samples from 20 prostate cancer patients was obtained from the GEO database (GSE111177) [25]. For immunotherapy, the data of 18 metastatic castration-resistant prostate cancer (mCRPC) patients treated with ipilimumab were obtained from the work of Subudhi et al. (EGAS00001004050 at the European Genome-phenome Archive) [26].

In total, 1158 prostate cancer patients were included in this study. The detailed baseline characteristics are demonstrated in Additional file 1: Table S1. In microarray datasets, probes were replaced with matched gene symbols in later analysis (the first one was selected if multiple genes were matched), while the probes that did not match any gene were discarded. For all datasets, multiple rows with the same gene symbol were ordered by the median expression value, and the max one was selected for further analysis.

### Prognosis-related markers selection and the development of the TICS algorithm

In total, 92 immune-related signatures were obtained from a previous work [27], and the single sample gene set enrichment analysis (ssGSEA) algorithm was applied to calculate the enrichment score (ES) of these immune-related signatures in the above cohorts. Based on the expression of all genes, the normalized Z-score value of mRNA expression profiles was used to calculate the score. Each signature was transformed into binary variables with the cutoff of median value in four training datasets (GSE46602, GSE54460, GSE70769, and GSE94767). The univariable Cox regression analysis was applied to identify the prognostic efficacy of ES stratification in each training set, then the meta-analysis was performed to estimate the hazard ratios (HRs) of four training sets for each signature. The signatures significantly associated with prognosis (*P* < 0.05) in the meta-analysis were selected to develop the TICS. The TICS was calculated by the following formula:

$$TICS=\sum_{i=1}^{n}(\frac{{1-HR}_{i}}{SE(HR)}*ssGSEA\,score)$$

where *HR*_*i*_ was the hazard ratio of *i*th signature from meta-analysis, and *ssGSEA score* represented the enrichment score of the corresponding signature.

To validate the prognostic performance of the TICS, three datasets (GSE70768, DKFZ2018, and TCGA-PRAD) were used as independent validation cohorts. The robustness and independence of the TICS as a predictor of BCRFS were evaluated by subgroup analysis and univariable/multivariable Cox regression. The added value of TICS to traditional prognostic factors such as pre-treatment PSA and Gleason score was evaluated by comparing the prognostic utility of two models constructed based on multivariable Cox regression. The first model consisted of pT, pN, surgical margin, PSA, Gleason score, and the TICS (continuous variable), and the second model involved all except the TICS.

### Identification of biological characteristics related to the TICS

We compared genetic features between the high- and low-risk groups, including microsatellite instability (MSI) score, tumor mutation burden (TMB), tumor purity, and genomic scar signatures [28]. Meanwhile, the Kyoto Encyclopedia of Genes and Genomes (KEGG) gene set (c2.cp.kegg.v7.5.1.symbols.gmt) was downloaded from MSigDB to investigate the potential difference in the biological function between the high- and low-TICS subgroups using the gene set enrichment analysis (GSEA) method. Additionally, the ssGSEA analysis based on published mitogenic-related gene sets was used to quantify the relative abundance in the TCGA-PRAD dataset [29].

### Statistical analyses

All data analyses and graph plotting were conducted in R software (version 4.1.2), SPSS (version 20.0.0), and GraphPad Prism (version 8.0.2). The meta-analysis was performed by the “meta” R package. The Kaplan–Meier method was used to access the survival of different subgroups, and the log-rank (Mantel–Cox) test was used to measure the statistical significance. The univariable and multivariable Cox proportional hazards regression analyses were performed using the “ezcox” R package. Time-dependent receiver operating characteristic (tROC) curves, concordance index (C-index), and calibration curves were calculated by the “timeROC” package to evaluate the predictive power of the TICS. Mann–Whitney U test and Chi-square test were used to assess the statistical difference between the two groups. Spearman’s correlation analysis was used for correlational analyses. The “ComplexHeatmap” package was used to visualize the mutation landscape. Unless otherwise stated, *P* values of less than 0.05 were considered statistically significant.

## Results

### Construction of the scoring algorithm based on recurrence-associated immune signatures

**Fig. 1:**
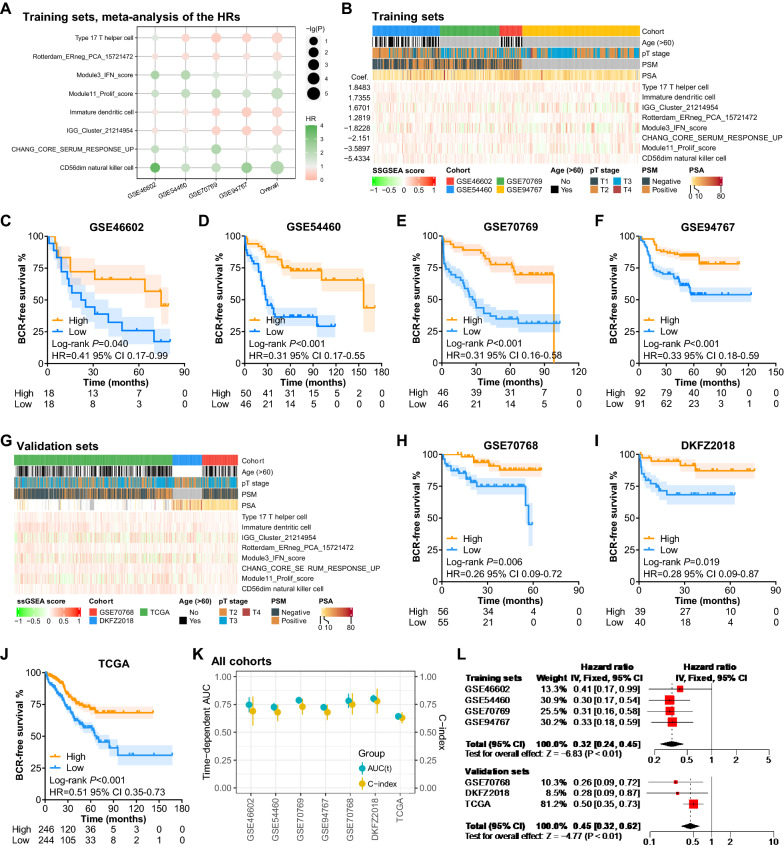
Construction and validation of the TICS. **A** Meta-analysis of the HRs of eight immune signatures. **B** Clinicopathological features and the expression pattern of prognostic immune signatures in training cohorts. **C**–**F** Kaplan–Meier analysis of the high- and low-TICS group in each training cohort. **G** Clinicopathological features and the expression pattern of immune signatures in validation cohorts. **H**–**J** Kaplan–Meier analysis of the high- and low-TICS group in each training cohort. **K** The time-dependent AUC and C-index of the TICS in each cohort. **L** The HR values of the TICS in training and validation cohorts by meta-analysis. (*PSM* positive surgical margin, *PSA* prostate specific antigen, *HR* hazard ratio, *BCR* biochemical recurrence, *CI* confidence interval, *TCGA* The Cancer Genome Atlas, *AUC* area under the curve.)

In total, 92 immune contexture-related signatures compiled in a previous study were investigated in our study and the genes in each signature are demonstrated in Additional file 1: Table S2. In the training cohorts (clinical information demonstrated in Additional file 1: Table S1), the association between BCRFS and the ssGSEA enrichment score of each signature as dichotomous variables according to the median value was analyzed. Then the mixed effects in the four training cohorts were calculated by meta-analysis. The prognostic effects of the 92 immune signatures in the training sets are demonstrated in Additional file 2: Fig. S1. Among these immune signatures, eight signatures showed a consistent association with BCRFS (Fig. 1A and Additional file 1: Table S3). In detail, the signatures related to type 17T helper cells, immature dendritic cells, cell death (Rotterdam_ERneg_PCA_15721472), and immunoglobulin (IGG_Cluster_21214954) heralded a long BCRFS (P < 0.05) [30, 31], while the signatures concerning CD56^dim^ natural killer (NK) cell, wound healing (CHANG_CORE_SERUM_RESPONSE_UP), interferon signaling (Module3_IFN_score), and proliferation (Mudule11_Prolif_score) were associated with a poor BCRFS (P < 0.05) [32, 33]. The ssGSEA enrichment score for the eight signatures are demonstrated in Fig. 1B, showing comparable scores between the training cohorts. We also checked the overlap of the genes involved in these eight signatures and only 11 genes were repeated in the signatures of CHANG_CORE_SERUM_RESPONSE_UP (212 genes) and Mudule11_Prolif_score (120 genes). In addition, low multicollinearity was observed among the scores of these eight signatures (variance inflation factor [VIF] < 10 and kappa < 100, Additional file 1: Table S4), which infers the feasibility of constructing a prognostic model based on these eight signatures.

According to the HR values of the mixed effects, we calculated the coefficients of the eight signatures (shown on the left in Fig. 1B) and calculated the TICS by summing the products of each signature score and its coefficient. According to the TICS, samples were divided into two groups with the median value in each training cohort, and the patients in the high-TICS group had a more favorable BCRFS than the corresponding low-TICS group (GSE46602: *P* = 0.040, HR = 0.41, 95% CI 0.17–0.99; GSE54460: *P* < 0.001, HR = 0.31, 95% CI 0.17–0.55; GSE70769: *P* < 0.001, HR = 0.31, 95% CI 0.16–0.58; GSE94767: *P* < 0.001, HR = 0.33, 95% CI 0.18–0.59; Fig. 1C–F).

To further evaluate the prognostic power of the TICS, three additional independent cohorts (GSE70768, DKFZ2018, and TCGA-PRAD) were utilized for validation (clinical information demonstrated in Additional file 1: Table S1). The ssGSEA enrichment score for the eight signatures are also demonstrated in Fig. 1G. Among these cohorts, the two groups divided by the median value of the TICS also showed significant differences in BCRFS (GSE70768: *P* = 0.006, HR = 0.26, 95% CI 0.09–0.72; GSE54460: *P* = 0.019, HR = 0.28, 95% CI 0.09–0.87; TCGA: *P* < 0.001, HR = 0.51, 95% CI 0.35–0.73; Fig. 1H–J). The AUCs were 0.64–0.80 and the c-indices were 0.63–0.78 for all the included cohorts (Fig. 1K and Additional file 1: Table S5), revealing that the TICS was likely to be a robust prognostic marker for the BCRFS after RP. Pooled analysis revealed consistent results in the training cohorts (HR = 0.32, 95% CI 0.24–0.45, *P* < 0.001) and in the validation cohorts (HR = 0.45, 95% CI 0.32–0.62, *P* < 0.001, Fig. 1L). Altogether these results suggest the robust utility of the TICS in predicting biochemical recurrence in prostate cancers.

Furthermore, we compared the TICS with several previously published multi-gene signatures with exposed formulas [34–37]. In the three datasets in the validation set, the TICS showed a numerically higher C-index for predicting BCRFS than other signatures (Additional file 1: Table S6 and Additional file 2: Fig. S2), elucidating the superiority of the TICS over previously published signatures.

### The added value of the TICS for prognostication in prostate cancer

**Fig. 2:**
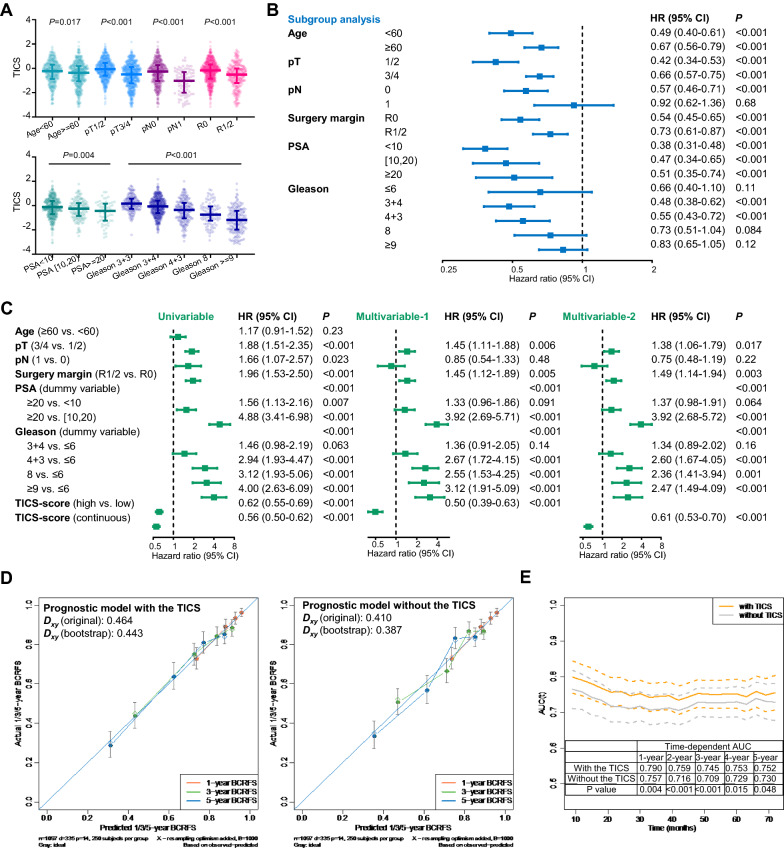
Added value of the TICS for prognostication in prostate cancer. **A** The distribution of the TICS in each clinicopathological subgroup and the correlation of the TICS with patients’ age/PSA level. **B** Subgroup analysis of the prognostic efficacy of the TICS. **C** Prognostic effect of the TICS in univariable and multivariable models. **D** The calibration curves of prognostic models with/without the TICS. **E** The time-dependent AUC of prognostic models with/without the TICS. (*PSA* prostate specific antigen, *HR* hazard ratio, *BCRFS* biochemical recurrence-free survival, *CI* confidence interval, *TCGA* The Cancer Genome Atlas, *AUC* area under the curve.)

We then assessed the associations between the TICS and clinicopathological features. The patients of all cohorts were grouped according to clinicopathological features, and the TICS were compared among different subgroups. A lower TICS was observed in elderly patients (≥ 60, *P* = 0.017), patients with advanced disease (pT3/4, *P* < 0.001; pN1, *P* < 0.001), patients with positive surgical margin (R1/2, *P* < 0.001), a higher pre-treatment prostate-specific antigen (PSA, *P* = 0.004), and a poorer Gleason score (*P* < 0.001, Fig. 2A).

Given the association between the TICS and prognostic indicators such as pathological stage, pre-treatment PSA, and Gleason score, we further performed a subgroup analysis of the association between the TICS and BCRFS. In the subgroups stratified by age, stage, surgical margin, PSA, and Gleason score, similar trends of the association between a high TICS and long BCRFS were observed (Fig. 2B), indicating the robustness of its prognostic utility. Furthermore, multivariable Cox regression analysis identified the TICS as an independent predictor of BCRFS, whether the TICS was treated as a categorical variable (grouped by the median value, multivariable HR = 0.50, 95% CI 0.39–0.63, *P* < 0.001) or as a continuous variable (multivariable HR = 0.61, 95% CI 0.53–0.70, *P* < 0.001, Fig. 2C), suggesting that the TICS was a prognostic factor independent of clinicopathological features.

We constructed two models by multivariable regression with or without the TICS to estimate the added value of the TICS. The first model consisting of pT, pN, surgical margin, PSA, Gleason score, and the TICS (continuous variable) achieved a bootstrap *D*_*xy*_ of 0.443. By contrast to this model, the second model involving all the above variables except the TICS yielded a relatively lower bootstrap *D*_*xy*_ of 0.387. The calibration curves of these two models are shown in Fig. 2D, in which the model with the TICS showed better agreement between the predicted and observed 1-, 3-, and 5-year BCRFS. Moreover, the addition of the TICS significantly improved the 1-year (0.790 vs. 0.757, *P* = 0.004), 2-year (0.759 vs. 0.716, *P* < 0.001), 3-year (0.745 vs. 0.709, *P* < 0.001), 4-year (0.753 vs. 0.729, *P* = 0.015), and 5-year AUC (0.752 vs. 0.730, *P* = 0.048, Fig. 2E). Adding the TICS into the model constructed by traditional prognostic markers markedly improved its prognostic utility, indicating the added value of the TICS for the prognostication based on the traditional indicators.

### Genetic profiles of the prostate cancer in different TICS groups

**Fig. 3:**
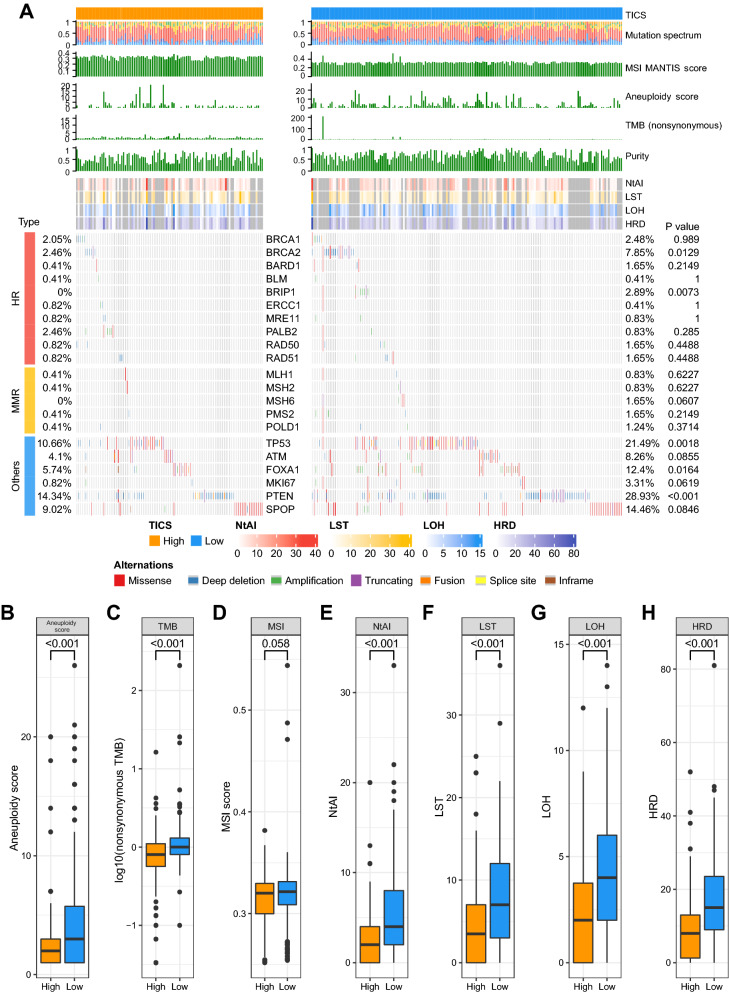
Mutational correlates of the TICS. **A** The genetic alterations of patients in the TCGA cohorts. **B**–**I** Comparison of genetic features between the high- and low-TICS groups (*MSI* microsatellite instability, *TMB* tumor mutation burden, *NtAI* telomeric allelic imbalance, *LST* large-scale state transitions, *LOH* loss of heterozygosity, *HRD* homologous recombination deficiency, *HR* homologous recombination genes, *MMR* mismatch repair genes)

Based on the TCGA dataset, we analyzed the somatic alterations of the cancerous samples in the high- and low-TICS groups (Fig. 3A). The frequency of the somatic mutations of homologous recombination (HR) genes and mismatch repair (MMR) genes trended higher in the low-TICS group (e.g., *BRCA2* [P = 0.013], *BRIP1* [P = 0.007], and *MSH6* [P = 0.062], Fig. 3A). In addition, somatic alterations in other key genes were also investigated (Fig. 3A). *TP53*, *FOXA1,* and *PTEN* were reported to be involved in the regulation of cell survival and proliferation, and their somatic mutations were associated with cancer progression in prostate cancer [38–40]. In our study, mutation events of these three genes were significantly increased in the low-TICS group (*TP53*, *P* = 0.002; *FOXA1*, *P* = 0.016; *PTEN*, *P* < 0.001). Other genes associated with tumorigenesis of prostate cancer, such as *ATM*, *MKI67*, and *SPOP* [39, 41–44], showed non-significant increases in somatic mutation frequency in the low-TICS group.

As for the indices concerning genomic alterations, the low-TICS group had a high aneuploidy score (*P* < 0.001, Fig. 3B) and non-synonymous TMB (*P* < 0.001, Fig. 3C), indicating genomic instability. Based on the approval of poly-ADP ribose polymerase inhibitor (PARPi) in prostate cancer, we further analyzed the scores related to DNA damage repair. Consistent with the numerically more mutations of MMR and HR genes in the low-TICS group, the samples with a low TICS trended to have a high MSI score (*P* = 0.058, Fig. 3D) and high homologous recombination deficiency (HRD)-associated scores including telomeric allelic imbalance (NtAI, *P* < 0.001, Fig. 3E), large-scale state transitions (LST, *P* < 0.001, Fig. 3F), loss of heterozygosity (LOH, *P* < 0.001, Fig. 3G), and the total HRD score (*P* < 0.001, Fig. 3H).

### The TICS represented alterations of cancer-related pathways

**Fig. 4:**
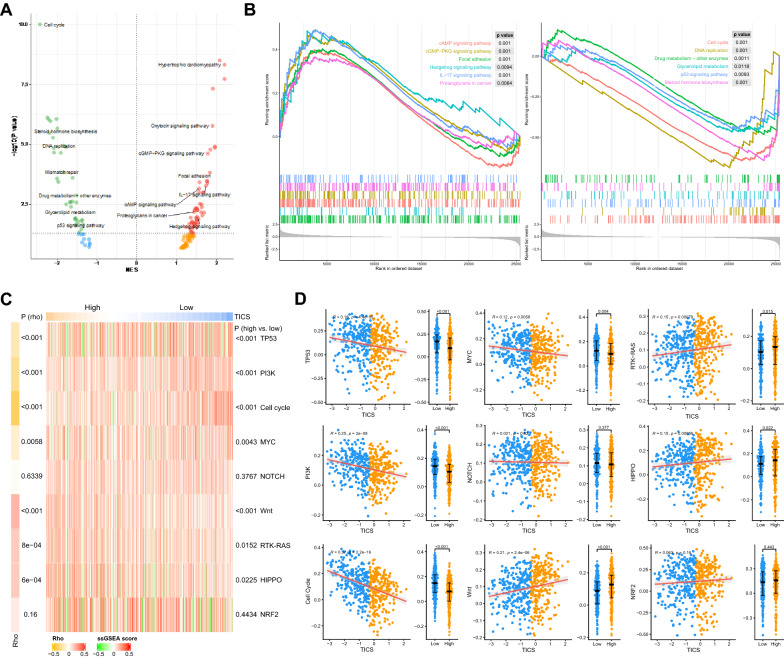
Molecular correlates of the TICS. **A** Volcanic map of the enrichment scores of KEGG pathways in the high- and low-TICS groups. **B** GSEA analysis of the significantly different cancer-related pathways. **C** Comparison of the ssGSEA score of mitogenic-related signatures in the high- and low-TICS patients in TCGA cohorts. **D** Correlation of the TICS with ssGSEA score of mitogenic-related signatures. (*NES* normalized enrichment score, *ES* enrichment score.)

Based on the gene sets embodied in the KEGG database, we analyzed the changes in pathway activity between the high- and low-TICS groups by GSEA (Fig. 4A and Additional file 1: Table S7). Tumors in the high-TICS group had higher activity of the Hedgehog signaling, second messenger signaling, and cell–matrix adhesion-related pathways, while the high expression of the IL-17 family may indicate an intense inflammatory response (Fig. 4B). On the other side, the abnormal expression of genes involved in DNA replication and cell cycle in the low-TICS group suggested the endogenous aggressiveness of tumors, and the activation of steroid hormone biosynthesis and drug metabolism may lead to resistance to ADT and other treatments (Fig. 4B).

Further, we analyzed the alterations of the previously reported oncogenic signaling pathways by Sanchez-Vega et al. [29]. The TICS was negatively correlated with the scores related to cell cycle (Rho = − 0.37, *P* < 0.001), p53 (Rho = − 0.37, *P* < 0.001), PI3K (Rho = − 0.25, *P* < 0.001), and Myc (Rho = − 0.12, *P* = 0.0058) pathways while positively correlated with the scores concerning Wnt (Rho = 0.21, *P* < 0.001), RTK/RAS (Rho = 0.15, *P* < 0.001), and Hippo (Rho = 0.15, *P* < 0.001) pathways (Fig. 4C, D). These results suggest that the TICS may represent the biological heterogeneity among different prostate cancers.

### Associations between TICS and the efficacy of ADT and immunotherapy

**Fig. 5:**
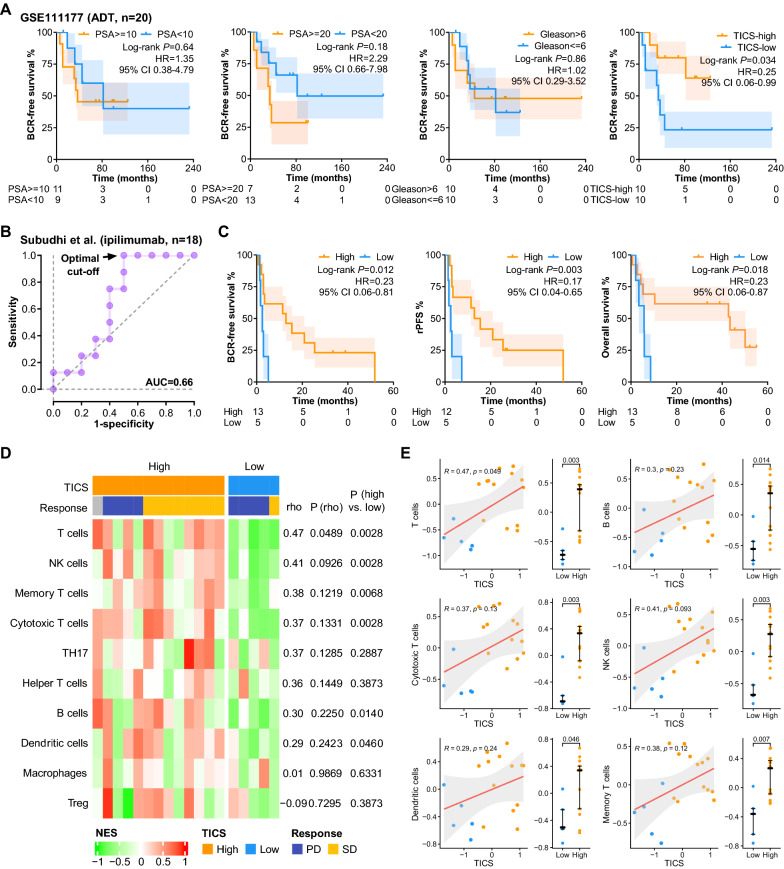
The associations of TICS with the efficacy of ADT and immunotherapy. **A** Kaplan–Meier analysis of the BCRFS of ADT patients (GSE111177) grouping with PSA level, Gleason score, or the TICS. **B** ROC curve for predicting patient response to immunotherapy based on the TICS. **C** Kaplan–Meier analysis of the BCRFS/rPFS/OS of the high- and low-TICS patients with immunotherapy. **D** The immune cell composition of the high- and low-TICS patients with immunotherapy. **E** Correlation of the immune cell fraction and the TICS of patients with immunotherapy. (*ADT* androgen deprivation therapy, *PSA* prostate specific antigen, *HR* hazard ratio, *BCR* biochemical recurrence, *CI* confidence interval, *AUC* area under the curve, *rPFS* radiographic progression-free survival.)

Considering the association between the TICS and steroid hormone biosynthesis pathway, we hypothesized that the TICS might predict the response to ADT. To evaluate the relationship between the TICS and ADT efficacy, an independent cohort (GSE111177) of 20 patients with clinical stage Ic to IIIa prostate cancer was analyzed (Fig. 5A). Neither pre-treatment PSA (< 10 vs. ≥ 10, or < 20 vs. ≥ 20) nor Gleason score (≤ 6 vs. > 6) was able to distinguish BCRFS effectively, while the TICS-based dichotomy based on median value yielded two groups with significantly different BCRFS (*P* = 0.034, HR = 0.25, 95% CI 0.06–0.99; Fig. 5A). Taken together, the TICS rather than pre-treatment PSA and Gleason score might be an effective marker to identify patients susceptible to castration resistance to ADT.

As a score reflecting immune contexture, the TICS should be theoretically associated with immunotherapy efficacy, which was studied in a cohort consisting of 18 CRPC patients treated with ipilimumab monotherapy [26]. First, based on the ROC curve illustrating the association between the TICS and disease control (Fig. 5B), an optimal cut-off was determined when the Youden’s index reached the maximum. According to this cut-off, the cohort was stratified into the high-TICS (n = 13) and the low-TICS groups (n = 5). Patients in the high-TICS group had significantly favorable BCRFS (*P* = 0.012, HR = 0.23, 95% CI 0.06–0.81), radiographic progression-free survival (rPFS, *P* = 0.003, HR = 0.17, 95% CI 0.04–0.65), and overall survival (OS, *P* = 0.018, HR = 0.23, 95% CI 0.06–0.87) compared to the low-TICS patients (Fig. 5C). We also analyzed the immune cell composition in the tumor tissues between both groups and found that the tumors in the high-TICS group were infiltrated with more T cells (*P* = 0.003, particularly cytotoxic T cells [*P* = 0.003] and memory T cells [*P* = 0.007]), B cells (*P* = 0.014), NK cells (*P* = 0.003) and dendritic cells (*P* = 0.046, Fig. 5D, E). These results indicate that the tumors with a low TICS may undergo the “immune desert” state, which is difficult to benefit from ICIs.

## Discussion

The recurrence of prostate cancer after RP is a serious threat, and the precise prediction of the risk of recurrence may facilitate appropriate personalized management. In the present study, we analyzed the expression profile of resected tumor samples and constructed a novel score to predict BCRFS based on the eight expression signatures related to immune contexture. The TICS was effective across the four training sets and the three validation sets and showed consistent prognostic effects in the subgroups stratified by key clinicopathological characteristics (e.g., TNM stage, pre-treatment PSA, and Gleason score). The TICS was further identified to be an independent predictor of BCRFS. Based on the prognostic model constructed by key clinicopathological features, adding the TICS significantly improved the power of prognostication. Moreover, the TICS may represent intrinsic discrepancies of prostate cancers at genomic and transcriptomic levels and possess the ability to predict the efficacy of ADT and ICI treatment.

In previous studies, the biological functions related to some of the signatures involved in the TICS calculation have been suggested to be associated with the prognosis. Inflammation mediated by Th17 was correlated with a low Gleason score [14], while the infiltration of dendritic cells conferred improved distant metastasis-free survival of patients [45, 46]. Prostate cancer at the onset of hormone resistance revealed an increased proliferative rate and a decreased apoptotic rate compared with hormone-responsive cancer [47], and the expression of genes involved in cell cycle progression was useful to predict biochemical recurrence [48]. Nevertheless, there are also some signatures involved in the TICS that had inconsistent results with previous studies. Low NK cell activity was associated with positive surgical margins in 51 postoperative patients [49], while highly effective NK cells revealed a good prognosis in a study including 18 patients with metastatic cancer [50], but the high expression of CD56^dim^ NK cell signature led to a decline of the TICS. Interferon-γ may suppress the invasive capacity of prostate cancer cells in vitro [51], and a low interferon-β level was an indicator to distinguish high-risk patients with localized cancer [52], but the Module3_IFN_score signature was negatively correlated with the TICS. These abnormalities may be related to the spatial and temporal distribution of cells with corresponding expression signatures in prostate cancer tissue, which will be elucidated in further studies.

Tumor stage, PSA level, and Gleason score have been used for prostate cancer risk stratification and are widely recommended in guidelines [53, 54]. However, each feature has its disadvantages, such as poor specificity [55], interobserver variation, and sampling problems [56], resulting in different manifestations among patients in the same risk group. The TICS in the present study predicted different outcomes in patients with similar TNM stage, PSA level, or Gleason score, and demonstrated its independence in multivariable analysis, indicating its ability as a novel prognostic biomarker. The prognostic model with the TICS performed better than the model constructed by clinicopathological features alone, suggesting the added value of the TICS to the existing risk stratification system. In addition, the TICS showed better performance compared with previously published models [34–37], indicating its added value.

Although existing studies have identified several factors associated with ADT response (such as the expression of PTEN or specific miRNAs) [57, 58], a clinical prognostic system to predict the outcomes of ADT is absent to date. ADT may cause remodeling of the immune microenvironment in prostate cancer and affect the patient's relapse-free survival [59], which inspired us to try to predict patients’ response to ADT with the TICS. The TICS demonstrated better prognostic efficacy than the PSA level or the Gleason score in a limited cohort, and this encouraging result needs to be validated on a larger scale. In previous clinical trials, few of the unselected patients benefited from treatment with ICIs, while several generally acknowledged immunotherapy biomarkers (i.e., high TMB, alterations in DNA damage repair [DDR] genes, or high-expression PD-L1) yielded controversial results in the different trials [26, 60–62]. Patients with low TICS revealed poor outcomes in the investigated cohort with CTLA-4 antibody treatment, while TMB or DDR gene mutations failed to make effective discrimination, suggesting that TICS may have a unique value in excluding patients who may not benefit from immunotherapy.

In our study, the transcriptomic data were from different platforms, which may lead to potential adverse effects on data analysis and the generalizability of results. Despite the lack of batch correction in our study, we did the following to minimize this disadvantage in developing a robust TICS. First, the seven PRAD datasets were split into the training set and the validation set based on platforms, ensuring that the training and validation sets each have datasets using microarray and RNA-seq. Second, the TICS was calculated by the rank-based ssGSEA method, suitable for cross-platform transcriptome measurements. In the xCell study using ssGSEA to estimate immune cell infiltration, the authors showed that their scores reliably predicted enrichment when using different RNA-seq techniques and different microarray platforms [63]. The xCell scores were agnostic to normalization methods or concerns related to batch effects, making them robust to both technical and biological noise [63]. Third, in the training set, the signature scores of different datasets were not directly combined. Instead, the prognostic value of each signature in each dataset independently was assessed by comparing the BCR of two subgroups classified by the median value of each signature in each dataset. Fourth, in model training, the coefficient of each signature score in the algorithm of the TICS was decided by the pooled HR by meta-analysis, reflecting the combined effect among the four datasets in the training set using different platforms. In validation, the TICS showed great performance in each of the three datasets in the validation set, including one using microarray (GSE70768) and two using RNA-seq (DKFZ2018 and TCGA-PRAD). These results indicate the potential generalizability of the TICS across platforms.

As for other limitations, first, the size of the cohorts used to analyze the associations of the TICS with the efficacy of ADT or immunotherapy efficacy was small, and castration-resistant patients in the immunotherapy cohort may have molecular characteristics different from the training cohorts. Our findings based on these two cohorts had hypothesis-generating rather than hypothesis-testing value. More data on prostate cancer patients with non-RNA-seq or microarray techniques (e.g., protein expression level) are needed to evaluate the precision and robustness of the TICS model in the future. Second, the TICS was calculated by the ssGSEA method which requires the whole microarray or RNA-seq data. Despite that RNA-seq may benefit PRAD patients by detecting fusions (e.g., *NTRK*), large genomic rearrangements (e.g., *BRCA1/2*), and splice variants (e.g., AR-V7) that may guide targeted therapies, its low cost-effectiveness may prevent it from being widely used in clinical settings. Third, the TICS consists of eight immune signatures/cells, while exploring the spatial relationship between immune cells and cancer cells must be warranted in future studies. Fourth, established with retrospective datasets, more prospective studies are necessary to examine the clinical utility and cost-effectiveness of the TICS.

## Conclusions

In summary, our study identified the TICS as a robust biomarker based on the expression signatures in prostate cancer. In a retrospective study of multiple cohorts, TICS can optimize the accuracy of existing models for predicting BCR, while it is also associated with the outcomes of ADT or immunotherapy. In the PRAD patients after curative surgery with a normal PSA and/or a low Gleason score, those with a low TICS might benefit from radical treatment and close monitoring. In addition, the predictive value of the TICS for ADT and immunotherapy is worth further investigation. This work developed a robust and effective tool for improving the risk stratification scheme of prostate cancer patients, which may optimize post-operative management and personalized treatment for patients with prostate cancer in clinical practice.

## Supplementary Information

**Additional file 1: Table S1.** Clinical characteristics of prostate cancer patients. **Table S2.** Genes involved in the 92 immune contexture-related signatures in the study. **Table S3.** Identified signatures correlated with BCRFS in meta-analysis of training cohorts. **Table S4.** Multicollinearity test used by variance inflation factors (VIF) and kappa test for selected signatures in all cohorts. **Table S5.** The C-index and AUC value of training and validation cohort. **Table S6.** Comparison of the TICS and other prognostic signatures. **Table S7.** GSEA results of the KEGG pathways in the high- and low-TICS groups in TCGA cohort.

**Additional file 2: Fig. S1.** The prognostic effect of immune signatures in the training sets. **Fig. S2.** Comparison of the TICS and other prognostic signatures.

## Data Availability

The datasets generated and analysed during the current study are available in Gene-Expression Omnibus (GEO) at GSE46602, GSE54460, GSE70768, GSE70769, GSE94767, and GSE111177, and in TCGA at TCGA-PRAD. The data from DKFZ2018 that was analyzed in this study was obtained from cBioPortal at http://www.cbioportal.org/study/summary?id=prostate_dkfz_2018. The data of mCRPC patients treated with ipilimumab were obtained from the additional data files of Subudhi et al.

## Abbreviations

ADT: Androgen deprivation therapy
BCR: Biochemical recurrence
BCRFS: Biochemical recurrence-free survival
CRPC: Castration-resistant prostate cancer
GSEA: Gene set enrichment analysis
HRD: Homologous recombination deficiency
ICI: Immune checkpoint inhibitor
LOH: Loss of heterozygosity
LST: Large-scale state transitions
MSI: Microsatellite instability
NtAI: Number of telomeric allelic imbalance
PSA: Prostatic specific antigen
RP: Radical prostatectomy
ssGSEA: Single sample gene set enrichment analysis
TICS: Tumor immune contexture score
TIME: Tumor immune microenvironment

## References

[1] Sung H, Ferlay J, Siegel RL, Laversanne M, Soerjomataram I, Jemal A (2021). Global cancer statistics 2020: GLOBOCAN estimates of incidence and mortality worldwide for 36 cancers in 185 countries. CA Cancer J Clin.

[2] Dubost C, Blondeau P, d’Allaines C, Piwnica A, Guilmet D (1965). Surgical treatment of tetralogy of fallot. (Complete correction under extracorporeal circulation). Arch Mal Coeur Vaiss.

[3] Li F, Mahato RI (2014). MicroRNAs and drug resistance in prostate cancers. Mol Pharm.

[4] Feng Q, He B (2019). Androgen receptor signaling in the development of castration-resistant prostate cancer. Front Oncol.

[5] Freeman JW (2021). Structural biology of the tumor microenvironment. Adv Exp Med Biol.

[6] Hinshaw DC, Shevde LA (2019). The tumor microenvironment innately modulates cancer progression. Cancer Res.

[7] Molavi O, Ma Z, Hamdy S, Lavasanifar A, Samuel J (2009). Immunomodulatory and anticancer effects of intra-tumoral co-delivery of synthetic lipid A adjuvant and STAT3 inhibitor, JSI-124. Immunopharmacol Immunotoxicol.

[8] Jafari S, Molavi O, Kahroba H, Hejazi MS, Maleki-Dizaji N, Barghi S (2020). Clinical application of immune checkpoints in targeted immunotherapy of prostate cancer. Cell Mol Life Sci.

[9] Flammiger A, Bayer F, Cirugeda-Kühnert A, Huland H, Tennstedt P, Simon R (2012). Intratumoral T but not B lymphocytes are related to clinical outcome in prostate cancer. APMIS.

[10] McArdle PA, Canna K, McMillan DC, McNicol AH, Campbell R, Underwood MA (2004). The relationship between T-lymphocyte subset infiltration and survival in patients with prostate cancer. Br J Cancer.

[11] Knutson KL, Disis ML (2005). Tumor antigen-specific T helper cells in cancer immunity and immunotherapy. Cancer Immunol Immunother.

[12] Yang W, Chen X, Hu H (2020). CD4+ T-cell differentiation in vitro. Methods Mol Biol.

[13] Zhu J, Yamane H, Paul WE (2010). Differentiation of effector CD4+ T cell populations. Annu Rev Immunol.

[14] Sfanos KS, Bruno TC, Maris CH, Xu L, Thoburn CJ, Demarzo AM (2008). Phenotypic analysis of prostate-infiltrating lymphocytes reveals T H17 and Treg skewing. Clin Cancer Res.

[15] Zhang Q, Liu S, Ge D, Cunningham DM, Huang F, Ma L (2017). Targeting Th17-IL-17 pathway in prevention of micro-invasive prostate cancer in a mouse model. Prostate.

[16] Hellmann MD, Paz-Ares L, Bernabe Caro R, Zurawski B, Kim S-W, Carcereny Costa E (2019). Nivolumab plus ipilimumab in advanced non-small-cell lung cancer. N Engl J Med.

[17] Isaacsson Velho P, Antonarakis ES (2018). PD-1/PD-L1 pathway inhibitors in advanced prostate cancer. Expert Rev Clin Pharmacol.

[18] Antonarakis ES, Piulats JM, Gross-Goupil M, Goh J, Ojamaa K, Hoimes CJ (2020). Pembrolizumab for treatment-refractory metastatic castration-resistant prostate cancer: multicohort, open-label phase II KEYNOTE-199 study. J Clin Oncol.

[19] Gao Z, Tao Y, Lai Y, Wang Q, Li Z, Peng S (2020). Immune cytolytic activity as an indicator of immune checkpoint inhibitors treatment for prostate cancer. Front Bioeng Biotechnol.

[20] Mortensen MM, Høyer S, Lynnerup AS, Ørntoft TF, Sørensen KD, Borre M (2015). Expression profiling of prostate cancer tissue delineates genes associated with recurrence after prostatectomy. Sci Rep.

[21] Long Q, Xu J, Osunkoya AO, Sannigrahi S, Johnson BA, Zhou W (2014). Global transcriptome analysis of formalin-fixed prostate cancer specimens identifies biomarkers of disease recurrence. Cancer Res.

[22] Ross-Adams H, Lamb A, Dunning M, Halim S, Lindberg J, Massie C (2015). Integration of copy number and transcriptomics provides risk stratification in prostate cancer: a discovery and validation cohort study. EBioMedicine.

[23] Luca BA, Brewer DS, Edwards DR, Edwards S, Whitaker HC, Merson S (2018). DESNT: a poor prognosis category of human prostate cancer. Eur Urol Focus.

[24] Gerhauser C, Favero F, Risch T, Simon R, Feuerbach L, Assenov Y (2018). Molecular evolution of early-onset prostate cancer identifies molecular risk markers and clinical trajectories. Cancer Cell.

[25] Sharma NV, Pellegrini KL, Ouellet V, Giuste FO, Ramalingam S, Watanabe K (2018). Identification of the transcription factor relationships associated with androgen deprivation therapy response and metastatic progression in prostate cancer. Cancers (Basel).

[26] Subudhi SK, Vence L, Zhao H, Blando J, Yadav SS, Xiong Q (2020). Neoantigen responses, immune correlates, and favorable outcomes after ipilimumab treatment of patients with prostate cancer. Sci Transl Med.

[27] Thorsson V, Gibbs DL, Brown SD, Wolf D, Bortone DS, Ou Yang TH (2018). The immune landscape of cancer. Immunity.

[28] Marquard AM, Eklund AC, Joshi T, Krzystanek M, Favero F, Wang ZC (2015). Pan-cancer analysis of genomic scar signatures associated with homologous recombination deficiency suggests novel indications for existing cancer drugs. Biomark Res.

[29] Sanchez-Vega F, Mina M, Armenia J, Chatila WK, Luna A, La KC (2018). Oncogenic signaling pathways in The Cancer Genome Atlas. Cell.

[30] Wang Y, Klijn JG, Zhang Y, Sieuwerts AM, Look MP, Yang F (2005). Gene-expression profiles to predict distant metastasis of lymph-node-negative primary breast cancer. Lancet.

[31] Fan C, Prat A, Parker JS, Liu Y, Carey LA, Troester MA (2011). Building prognostic models for breast cancer patients using clinical variables and hundreds of gene expression signatures. BMC Med Genomics.

[32] Chang HY, Sneddon JB, Alizadeh AA, Sood R, West RB, Montgomery K (2004). Gene expression signature of fibroblast serum response predicts human cancer progression: similarities between tumors and wounds. PLoS Biol.

[33] Wolf DM, Lenburg ME, Yau C, Boudreau A, Van’t Veer LJ (2014). Gene co-expression modules as clinically relevant hallmarks of breast cancer diversity. PLoS ONE.

[34] Luan J, Zhang Q, Song L, Wang Y, Ji C, Cong R (2021). Identification and validation of a six immune-related gene signature for prediction of biochemical recurrence in localized prostate cancer following radical prostatectomy. Transl Androl Urol.

[35] Long G, Ouyang W, Zhang Y, Sun G, Gan J, Hu Z (2021). Identification of a DNA repair gene signature and establishment of a prognostic nomogram predicting biochemical-recurrence-free survival of prostate cancer. Front Mol Biosci.

[36] Wang X, Lv Z, Xia H, Guo X, Wang J, Wang J (2022). Biochemical recurrence related metabolic novel signature associates with immunity and ADT treatment responses in prostate cancer. Cancer Med.

[37] Wu X, Lv D, Lei M, Cai C, Zhao Z, Eftekhar M (2020). A 10-gene signature as a predictor of biochemical recurrence after radical prostatectomy in patients with prostate cancer and a Gleason score ≥7. Oncol Lett.

[38] Wang S, Gao J, Lei Q, Rozengurt N, Pritchard C, Jiao J (2003). Prostate-specific deletion of the murine Pten tumor suppressor gene leads to metastatic prostate cancer. Cancer Cell.

[39] Barbieri CE, Baca SC, Lawrence MS, Demichelis F, Blattner M, Theurillat JP (2012). Exome sequencing identifies recurrent SPOP, FOXA1 and MED12 mutations in prostate cancer. Nat Genet.

[40] Mu P, Zhang Z, Benelli M, Karthaus WR, Hoover E, Chen CC (2017). SOX2 promotes lineage plasticity and antiandrogen resistance in TP53-and RB1-deficient prostate cancer. Science (80−).

[41] Blattner M, Liu D, Robinson BD, Huang D, Poliakov A, Gao D (2017). SPOP mutation drives prostate tumorigenesis in vivo through coordinate regulation of PI3K/mTOR and AR SIGNALING. Cancer Cell.

[42] Fraser M, Sabelnykova VY, Yamaguchi TN, Heisler LE, Livingstone J, Huang V (2017). Genomic hallmarks of localized, non-indolent prostate cancer. Nature.

[43] Neeb A, Herranz N, Arce-Gallego S, Miranda S, Buroni L, Yuan W (2021). Advanced prostate cancer with ATM loss: PARP and ATR inhibitors. Eur Urol.

[44] Hammarsten P, Josefsson A, Thysell E, Lundholm M, Hägglöf C, Iglesias-Gato D (2019). Immunoreactivity for prostate specific antigen and Ki67 differentiates subgroups of prostate cancer related to outcome. Mod Pathol.

[45] Zhao SG, Lehrer J, Chang SL, Das R, Erho N, Liu Y (2019). The immune landscape of prostate cancer and nomination of PD-L2 as a potential therapeutic target. J Natl Cancer Inst.

[46] Zhang C, Chen T, Li Z, Liu A, Xu Y, Gao Y (2021). Depiction of tumor stemlike features and underlying relationships with hazard immune infiltrations based on large prostate cancer cohorts. Brief Bioinform.

[47] Quinn DI, Henshall SM, Sutherland RL (2005). Molecular markers of prostate cancer outcome. Eur J Cancer.

[48] Cuzick J, Swanson GP, Fisher G, Brothman AR, Berney DM, Reid JE (2011). Prognostic value of an RNA expression signature derived from cell cycle proliferation genes in patients with prostate cancer: a retrospective study. Lancet Oncol.

[49] Lu YC, Kuo MC, Hong JH, Jaw FS, Huang CY, Cheng JCH (2020). Lower postoperative natural killer cell activity is associated with positive surgical margins after radical prostatectomy. J Formos Med Assoc.

[50] Pasero C, Gravis G, Granjeaud S, Guerin M, Thomassin-Piana J, Rocchi P (2015). Highly effective NK cells are associated with good prognosis in patients with metastatic prostate cancer. Oncotarget.

[51] Creighton CJ (2007). A gene transcription signature associated with hormone independence in a subset of both breast and prostate cancers. BMC Genomics.

[52] Eiró N, Bermudez-Fernandez S, Fernandez-Garcia B, Atienza S, Beridze N, Escaf S (2014). Analysis of the expression of interleukins, interferon β, and nuclear factor-κ B in prostate cancer and their relationship with biochemical recurrence. J Immunother.

[53] Mohler JL, Antonarakis ES, Armstrong AJ, D’Amico AV, Davis BJ, Dorff T (2019). Prostate cancer, Version 2. 2019, NCCN clinical practice guidelines in oncology. J Natl Compr Cancer Netw.

[54] Schaeffer E, Srinivas S, Antonarakis ES, Armstrong AJ, Bekelman JE, Cheng H (2021). NCCN guidelines insights: prostate cancer, Version 1. 2021. J Natl Compr Cancer Netw.

[55] Malik A, Srinivasan S, Batra J (2019). A new era of prostate cancer precision medicine. Front Oncol.

[56] Hughes C, Murphy A, Martin C, Sheils O, O’Leary J (2005). Molecular pathology of prostate cancer. J Clin Pathol.

[57] Mithal P, Allott E, Gerber L, Reid J, Welbourn W, Tikishvili E (2014). PTEN loss in biopsy tissue predicts poor clinical outcomes in prostate cancer. Int J Urol.

[58] Konoshenko MY, Bryzgunova OE, Laktionov PP. miRNAs and androgen deprivation therapy for prostate cancer. Biochim. Biophys. Acta - Rev. Cancer. 2021. 10.1016/j.bbcan.2021.188625 34534639

[59] Long X, Hou H, Wang X, Liu S, Diao T, Lai S (2020). Immune signature driven by ADT-induced immune microenvironment remodeling in prostate cancer is correlated with recurrence-free survival and immune infiltration. Cell Death Dis.

[60] Cha HR, Lee JH, Ponnazhagan S (2020). Revisiting immunotherapy: a focus on prostate cancer. Cancer Res.

[61] Graff JN, Beer TM, Alumkal JJ, Slottke RE, Redmond WL, Thomas GV (2020). A phase II single-arm study of pembrolizumab with enzalutamide in men with metastatic castration-resistant prostate cancer progressing on enzalutamide alone. J Immunother Cancer.

[62] Fizazi K, González Mella P, Castellano D, Minatta JN, Rezazadeh Kalebasty A, Shaffer D (2022). Nivolumab plus docetaxel in patients with chemotherapy-naïve metastatic castration-resistant prostate cancer: results from the phase II CheckMate 9KD trial. Eur J Cancer.

[63] Aran D, Hu Z, Butte AJ (2017). xCell: digitally portraying the tissue cellular heterogeneity landscape. Genome Biol.

