# Supplementary material for: Tumor immune contexture predicts recurrence after prostatectomy and efficacy of androgen deprivation and immunotherapy in prostate cancer
Source: J Transl Med. 2023 Mar 14;21:194. doi: 10.1186/s12967-022-03827-4 (PMC10012744; doi:10.1186/s12967-022-03827-4)

Additional file 2: Fig. S1. The prognostic effect of immune signatures in the training sets

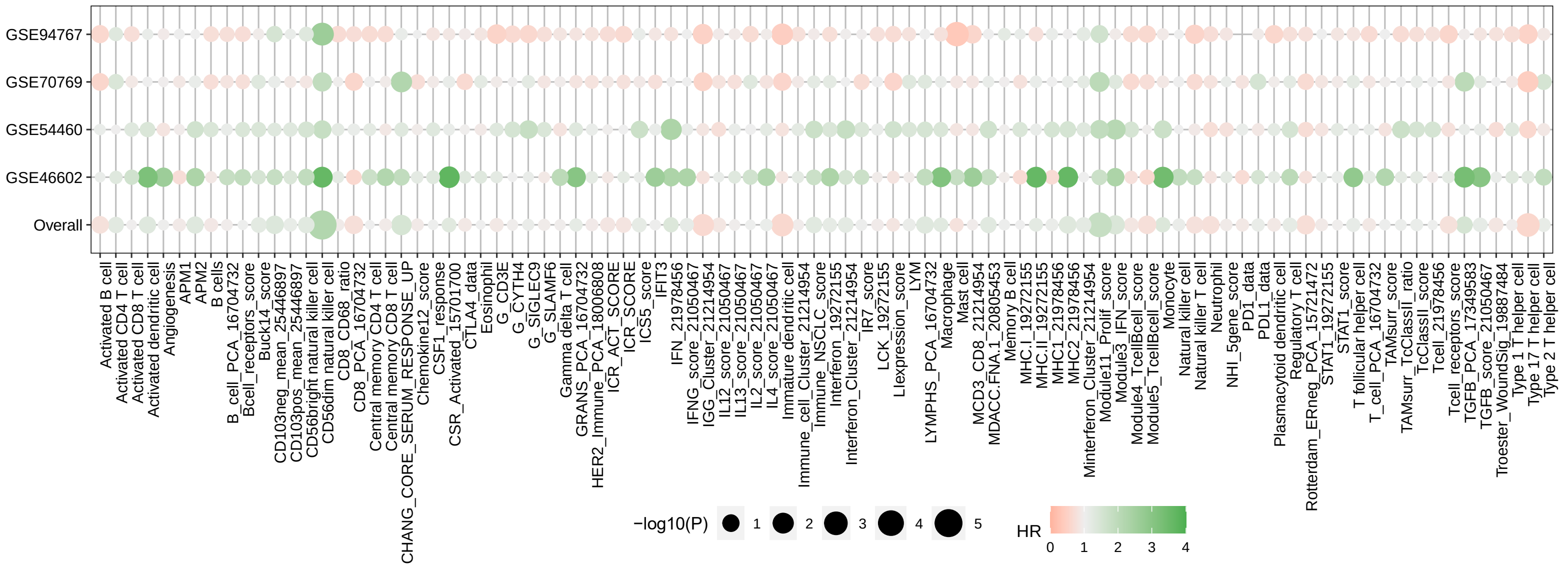

Additional file 2: Fig. S2. Comparison of the TICS and other prognostic signatures

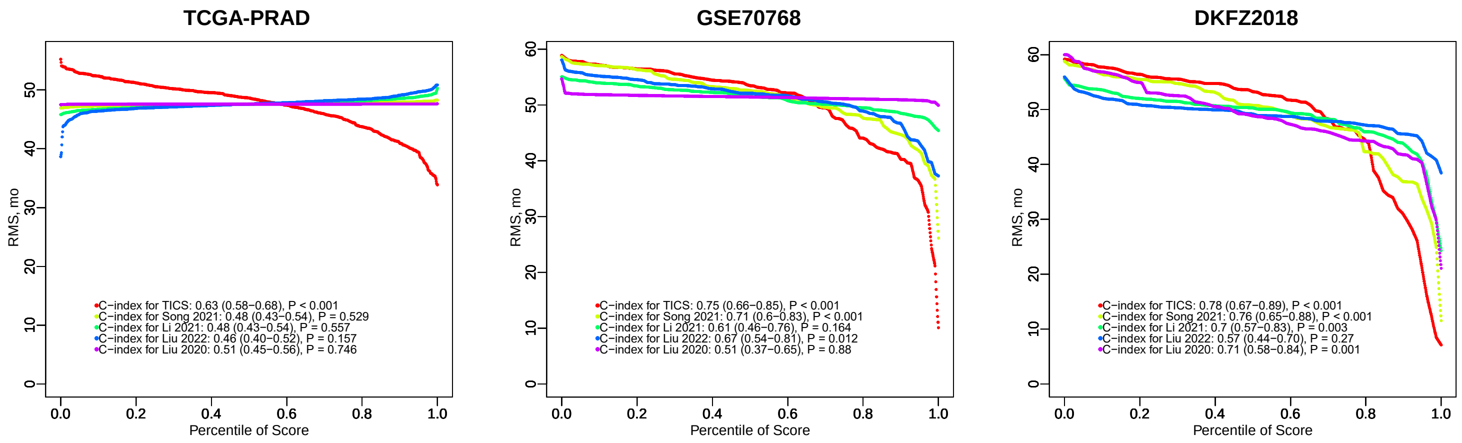

Supplement: Supplementary file 2 — Additional file 2: Fig. S1. The prognostic effect of immune signatures in the training sets. Fig. S2. Comparison of the TICS and other prognostic signatures. [file 12967_2022_3827_MOESM2_ESM.pdf]
